# Case Report: Personalized therapy in gynecological neuroendocrine neoplasm: a case of BRCA2-mutated ovarian neuroendocrine carcinoma treated with PARP-inhibitor

**DOI:** 10.3389/fonc.2026.1774835

**Published:** 2026-08-04

**Authors:** Mara Mantiero, Maggie Polignano, Anna Bassetti, Simone Oldani, Roberta Serino, Alessandra Russo, Cecilia Villa, Monika Ducceschi, Francesco Raspagliesi

**Affiliations:** 1 Gynecologic Oncology Unit, Fondazione IRCCS Istituto Nazionale dei Tumori, Milano, Italy; 2 Medical Oncology Department, Fondazione IRCCS Istituto Nazionale dei Tumori, Milano, Italy

**Keywords:** BRCA, neuroendocrine tumor, O-NEC, ovarian cancer, PARP inhibitors

## Abstract

**Introduction:**

Neuroendocrine neoplasms (NENs) of the female genital tract are rare and typically aggressive malignancies, accounting for only 1-2% of gynecologic tumors. The majority (54%) are located in the cervix. Ovarian neuroendocrine carcinomas (O-NECs) represent less than 1% of all ovarian cancers and 16% of all gynecologic NENS; these tumors are associated with poor prognosis, with an overall survival (OS) ranging from 11 to 20 months and progression-free survival (PFS) between 12-14.71 months. To date, approximately 923 cases have been reported in the literature and owing to their rarity no standardized treatment protocols have been established.

**Materials and methods:**

We retrospectively reviewed all cases of NENs of female genital tract treated at the Gynecologic Oncology Unit of Fondazione IRCCS Istituto Nazionale Tumori of Milan, Italy. Moreover, we describe an interesting case of a 66-year-old woman with O-NEC and harboring a BRCA2 mutation. Patient achieved a pathological complete response following platinum-based chemotherapy and subsequently received a maintenance therapy with PARP-inhibitor.

**Results:**

From 2005 to June 2025, a total of 42 women with NENs of the female genital tract were treated at the Gynecologic Oncology Unit of Fondazione IRCCS Istituto Nazionale Tumori of Milan (Italy): 59% originated from cervix, 29% from endometrium and 12% from ovaries. Median OS and PFS of patients with O-NEC were consistent with literature data (15 and 11 months, respectively); all patients underwent surgery followed by platinum-etoposide chemotherapy and then follow up. In May 2024, a 66-year-old woman underwent exploratory laparoscopy for a large pelvic mass (121×83×137 mm) associated with hemoperitoneum, bilateral grade 3 hydronephrosis and rectum infiltration. Final histopathological diagnosis was of small cell ovarian neuroendocrine carcinoma (SCNEC), FIGO stage IV, due to the presence of the hepatic metastasis. Next-generation sequencing (NGS) performed on tumor tissue revealed a pathogenetic BRCA2 mutation. Following multidisciplinary tumor board discussion, patient received 4 cycles of carboplatin-etoposide chemotherapy with an excellent radiological response and significant tumor marker reduction (CA125: 477→9.4 U/ml; NSE: 629→8.9 ng/mL; CEA: 560→16.9 ng/mL). The histopathology report, performed after interval debulking surgery, confirmed a complete pathological response. She completed systemic treatment with two additional cycles of chemotherapy and then, she started compassionate-use maintenance therapy with PARP inhibitor based on the presence of the BRCA2 mutation. Today, after 12 months to the end of chemotherapy, patient has not evidence of recurrence disease, she is in good clinical condition and maintenance treatment with PARP inhibitor is still ongoing. To the best of our knowledge, only 1 prior case of BRCA2-mutated O-NEC patient treated with PARP-inhibitor has been reported in literature, with a PFS of 2 months, suggesting limited efficacy of PARP inhibitors in this setting. However, our case may indicate a potential benefit in selected patients.

**Conclusion:**

O-NECs are rare and poorly characterized malignancies, lacking standardized diagnostic and therapeutic protocols. Therefore, comprehensive characterization of individual cases is crucial to improve knowledge and patient outcomes. This case highlights the pivotal role of molecular profiling in identifying candidates for personalized therapeutic approaches. Further experiences are needed to expand current knowledge and evaluate the potential benefit of PARP inhibitors in BRCA-mutated O-NEC patients.

## Introduction

Neuroendocrine neoplasms (NENs) are a heterogeneous group of epithelial tumors arising from neuroendocrine cells distributed throughout multiple organ system (1). The gastrointestinal tract is the most common site of origin of NENs (62–70%), followed by the tracheobronchial system (25%), and genital tract (<2%) (2).

Well-differentiated neuroendocrine tumors (NET) and poorly differentiated neuroendocrine carcinomas (NEC) represent two different families of NENs. According to WHO classification (5^th^ edition), we distinguish in NET G1, G2 and G3 about morphology, mitotic index and Ki-67. Both NET and NEC express neuroendocrine markers (synaptophysin and chromogranin) in immunohistochemistry, but with different pattern. About molecular characteristics, NETs and NECs typically harbor distinct mutational profiles: NETs often present mutations in genes such as MEN1, DAXX, ATRX (especially in pancreatic NETs), while NECs are frequently characterized by co-alterations of TP53 and RB1 and additional genetic changes common in high-grade carcinomas (e.g., KRAS, BRAF) — alterations that are seldom found in NET (3).

## Ovarian NENs

NENs of the female genital tract are rare, accounting for only 1-2% of gynecological malignancies and are classified according to site origin: cervix (54%), uterus (24%), ovaries (16%), vagina (5%) and vulva (1%) (4). Primary ovarian NENs (O-NENs) are rare entities accounting for less than 1% of all ovarian malignancies and are characterized by aggressive behavior. According to the 2022 WHO classification, ovarian NENs are divided into two main groups by histologic grade: ovarian carcinoid tumors (Grade 1) and O-NEC (NECs, Grade 3).

Ovarian carcinoids are usually well-differentiated and slow-growing tumors, whereas ovarian neuroendocrine carcinomas (O-NECs) represent a markedly more aggressive subgroup. O-NECs are further stratified into small cell, non-small cell (or large cell), and carcinoma subtypes. Small-cell carcinoma of the ovary (SCCO) comprises two distinct histologic entities: the pulmonary type (SCCOPT) and the hypercalcemic type (SCCOHT) (1). Recent studies have demonstrated that SCCOHTs harbor pathogenic, inactivating mutations of the SMARCA4 gene. Because SMARCA4 alterations are typical of rhabdoid tumors, SCCOHT is now considered a malignant rhabdoid tumor of the ovary rather than a true neuroendocrine carcinoma. Immunohistochemically, SCCO typically exhibits positivity for at least one neuroendocrine marker such as chromogranin A (CgA), synaptophysin (Syn), neuron-specific enolase (NSE), or CD56. CgA is reported to be the most specific marker and CD56 emerges as the most sensitive (5). The most frequently reported prognostic factors for early-stage disease are FIGO stage, lymph node metastasis, parametrial invasion, tumor size, lymphovascular space involvement (LVSI), histological heterogeneity, older age and smoking. Among these, Ishikawa et al. identified LVSI as the most significant prognostic factor for both overall survival and disease-free survival whereas pelvic lymph node metastasis was the key prognostic factor for disease-free survival (6, 7).

## Case description

We describe the case of a 66-year-old woman who underwent in May 2024 surgery for a bleeding pelvic mass associated with pathological tissue spread in the Douglas pouch, mesorectum, and pelvic lymphadenopathies. Surgery was initiated with an exploratory laparotomy and subsequently converted to laparotomy. Given the inability to achieve optimal debulking, after control of the active hemorrhage, the patient was referred for systemic neoadjuvant therapy. Histology showed small-cell neuroendocrine carcinoma (Ki67 70%). Tumor markers were elevated (CA125: 477 U/ml; NSE: 629 ng/mL; CEA: 560 ng/mL); baseline CT scan revealed a 121×83×137 mm right pelvic mass, retro-/para-aortic lymphadenopathy (6 × 10 cm), and secondary lesions in liver segment VI (13 mm) and splenic hilum (22 mm). The right ureter was encased with grade III hydronephrosis, the left showed grade I–II hydronephrosis. Given the rarity of the disease and the limited therapeutic options, we sought to further investigate the diagnosis through molecular characterization using next-generation sequencing (NGS), with the aim of identifying potential therapeutic targets. This analysis revealed the presence of two variants with pathogenic or potentially pathogenic significance: BRCA2 (exon 14, c.7180A>T, amino acid variant p.Arg2394Ter [R2394], allele frequency 89%) and ARID2 (exon 15, c.3940del, amino acid variant p.Ser1314ValfsTer8 [S1314Vfs8], allele frequency 9%). Also TP53 and RB1 were mutated as commonly observed in this histological subtype. Following multidisciplinary tumor board discussion, from July 2024, the patient received four cycles of carboplatin–etoposide, with relevant reduction in tumor markers (CEA 16.9 ng/mL, CA125 9.4 U/mL, NSE 8.9 ng/mL) and a complete radiologic response at the CT scan. In October 2024, an optimal debulking surgery was subsequently carried (bilateral adnexectomy, infracolic omentectomy, splenectomy and multiple peritoneal biopsies) without micro or macroscopic residual disease at the end of surgery (RT = 0). The final histological report defines a complete pathological response in all samples: all tissue samples were characterized by necrosis and chronic xanthogranulomatous inflammation, consistent with post-therapeutic changes, with no evidence of residual neoplasia. Two further chemotherapy cycles with carboplatin-etoposide were completed, with no clinically relevant complications or toxicity. On December 2024, at the end of systemic therapy, thoracic and abdomen CT scan and tumor markers were negative for disease. The patient, therefore, demonstrated an excellent response to platinum-based therapy and was a carrier of a BRCA2 mutation; consequently, following compassionate use authorization, the patient was able to start oral maintenance treatment with a Poly(ADP-ribose) polymerase (PARP) inhibitor, highlighting the potential role of targeted therapies in selected patients with O-NEC.

At the time of writing, 12 months after completion of platinum-based-chemotherapy, patient is free of disease recurrence, in good clinical condition, and maintenance therapy with Niraparib 100 mg/die is still ongoing; a dose reduction was required at the second cycle of maintenance therapy due to drug-related grade 3 thrombocytopenia, without signs of bleeding.

We do not have information regarding the germline status of the BRCA mutation, as the patient declined germline genetic testing. With respect to family history, the patient’s father was the only family member who died from a malignancy (gastric cancer).

## Discussion

In a recent systematic review, approximately 923 cases O-NEC are collected and, owing to their rarity, no standardized treatment protocols have been established (1). Pang et al. conducted a study involving 431 patients diagnosed with ovarian neuroendocrine tumors, analyzing clinical outcomes according to disease stage and treatment approach.

In early-stage disease (I–II), patients treated with surgery alone showed excellent results, with 5-year OS and cancer specific survival (CSS) rates of about 97%. In contrast, those who received surgery plus concurrent chemoradiotherapy (CCRT) had lower survival (OS 65.5%, CSS 57.1%).

In advanced stages, patients treated with surgery + chemotherapy + radiotherapy achieved better survival (5-year OS 72.9%, CSS 70.0%) compared with those undergoing surgery alone (OS 17.5%, CSS 19.3%).

Overall, surgery alone was effective in early-stage disease, while multimodal therapy was necessary to improve outcomes in advanced cases (6). In clinical practice, management is generally adapted from guidelines for epithelial ovarian cancer: first-line management is generally based on surgical resection followed by systemic chemotherapy, most commonly with an etoposide–cisplatin regimen. In case of recurrence, treatment typically relies on second-line agents such as paclitaxel, irinotecan, or doxorubicin, often administered in combination with localized radiotherapy. Although recurrent tumors have not been consistently associated with actionable genetic alterations, emerging reports suggest a potential therapeutic role for novel targeted approaches—including mTOR inhibitors, PD-1 monoclonal antibodies, and anti-angiogenic agents. To date, only one case of BRCA2-mutated O-NEC treated with a PARP inhibitor has been previously reported (5); in that case, however, patient experienced recurrence after 2 months, suggesting PARP inhibitors may not be beneficial for patients with NEC of the ovary. However our experience with niraparib, appears to be different, with a benefit in disease control currently lasting 12 months and preservation of a good quality of life for the patient. This difference experience may be explained by a possible different BRCA2 mutation (the specific variant was not reported in the article by Xing XY et al.) or by a different degree of heterogeneity of the neuroendocrine tumor cells themselves.

The concomitant presence of BRCA2 and ARID2 mutations in the index case may suggest a higher degree of homologous recombination deficiency (HRD), potentially resulting in increased vulnerability to DNA-damaging agents. This molecular background could partly explain the remarkable therapeutic response observed in our patient, both to platinum-based chemotherapy and subsequent maintenance treatment with niraparib. Furthermore, the coexistence of these alterations raises the possibility that defects in multiple DNA repair pathways may have contributed to enhanced treatment sensitivity. Although this hypothesis cannot be confirmed on the basis of a single case, it provides a biologically plausible explanation for the durable clinical benefit observed and warrants further investigation in larger cohorts.

In our patient, in the event of systemic disease progression, a platinum rechallenge may be considered, given a good platinum free interval (12 months) and the excellent response achieved with first-line platinum-based treatment. A valid systemic alternative option could also be enrollment in an investigational clinical trial, if available and if patient meets eligibility criteria.

We perform a retrospective review of all cases of neuroendocrine neoplasms (NENs) of the female genital tract managed at the Gynecologic Oncology Unit of the Fondazione IRCCS Istituto Nazionale dei Tumori, Milan, Italy, from October 2005 to June 2025. During this period, we collect a total of 42 women with NENs of the female genital tract (**Table 1**): 59% originated from the cervix, 29% from the endometrium, and 12% from the ovaries. Most of patients underwent surgical management followed by systemic platinum–etoposide chemotherapy and subsequent follow-up; all patients with recurrence, died due to progression disease. No patients received target therapy or immunotherapy. Early stage disease at diagnosis (FIGO I–II) and absence of residual tumor post-surgery, are the most important prognostic factors for all the populations.

**Table 1:** NECs of female genital tract treated at Gynecologic Oncology Unit of Fondazione IRCCS Istituto Nazionale dei Tumori, Milan, Italy (From 2005 to 2025).

	Main characteristics		
Site of origin	N cervix (%)	N endometrium (%)	N ovaries (%)
Number of Patients	25 (59)	12 (29)	5 (12)
Median age	48	57	43
FIGO stage at diagnosis			
I-II III-IV	16 (64) 9 (36)	6 (50) 6 (50)	2 (40) 3 (60)
Debulking Surgery^@^	24 (96)	10 (84)	3 (60)
Post-Surgery RT			
RT=0 RT >0 RT= not available	18 (75) 0 6 (25)	9 (90) 1 (10) 0	3 (100) 0 0
Systemic CHT – I line	24 (96)	12 (100)	5 (100)
Platinum -Etoposide	24 (100)	12 (100)	5 (100)
Recurrence (%)	11 (61)°	5 (56)*	1 (33)^#^

Survival			
Live	7 (28)	4 (33.5)	3 (60)
Death	11 (44)	5 (41.5)	/
Lost at Follow-up	7 (28)	3 (25)	2 (40)
Median. OS	23 m	19 m	15 m
Median. PFS	17 m	12 m	11 m

NECs, Neuroendocrine Carcinomas; RT, Residual Tumor; CHT, chemotherapy; m, months.

°7 patients lost at follow up after I line CHT; *3 patients lost at follow up after I line CHT; ^#^2 patients lost at follow up after I line CHT; ^@^Patients with stage IV disease and systemic involvement did not undergo surgery.

Regarding O-NEC, median age at diagnosis was 43 year old, younger than median age of most common serous histotype; median OS and PFS of this population were 15 and 11 months, respectively, consistent with previously published data.

The case of our patient is of particular scientific interest due to the highly challenging disease presentation, characterized by an extremely high tumor burden, followed by an excellent clinical response after the initial cycles of chemotherapy. This pattern is relatively typical of neuroendocrine tumors treated with platinum and etoposide-based regimens. What makes this case unique is the opportunity to administer maintenance therapy in this setting, owing to the identification of a specific BRCA mutation. The diagnostic work-up was otherwise standard, with the exception of next-generation sequencing (NGS), which was exceptionally performed in this patient. The results of this analysis opened up novel and clinically relevant therapeutic perspectives.

## Conclusion

Ovarian neuroendocrine carcinomas remain an exceptionally rare and poorly defined entity, with no standardized diagnostic pathways or therapeutic guidelines currently available. For this reason, the detailed reporting of individual cases is of great value, as it contributes to expanding the limited body of knowledge and may help guide clinical practice in the absence of robust evidence. Our experience highlights how molecular profiling can play a decisive role in tailoring treatment strategies, allowing the identification of patients who might benefit from targeted approaches. In particular, the durable response observed in our BRCA2-mutated patient suggests that PARP inhibitors could represent a promising therapeutic option in selected patients. Nevertheless, additional clinical experiences and larger series are required to better define their potential role and to establish more effective treatment strategies for this challenging disease.

## Patient perspective

We obtain written informed consent from the patients for the publication of the clinical case.

## Data Availability

The original contributions presented in the study are included in the article/supplementary material. Further inquiries can be directed to the corresponding author.
